# Reduction of Pain After Laparoscopic Bariatric Surgery by Personalized Checkpoint Acupuncture—Data of a STRICTA Conform Pilot Study

**DOI:** 10.1007/s11695-023-06654-8

**Published:** 2023-05-29

**Authors:** Erfan Ghanad, Sophie Staff, Christel Weiß, Mario Goncalves, Maria Joao Santos, Nuno Correia, Georgi Vassilev, Florian Herrle, Christoph Reissfelder, Henry Johannes Greten, Mirko Otto, Cui Yang

**Affiliations:** 1 Heidelberg School of Chinese Medicine, 69126 Heidelberg, Germany; 2 Department of Surgery, Medical Faculty Mannheim, University Medicine Mannheim, University of Heidelberg, Theodor-Kutzer-Ufer 1-3, 68167 Mannheim, Germany; 3 Department of Medical Statistics and Biomathematics, Medical Faculty Mannheim, Heidelberg University, 68167 Mannheim, Germany; 4 Institute of Biomedical Sciences Abel Salazar, University of Porto, 4050-313 Porto, Portugal; 5 Institute Piaget, Gaia, 4405-678 Vila Nova de Gaia, Portugal

**Keywords:** Obesity, Analgesia, Postoperative pain, Complementary medicine, Acupuncture

## Abstract

**Background:**

It remains challenging in clinical practice to perform optimal pain management following bariatric surgeries. Acupuncture (AC) is an effective method of postoperative pain management, but its clinical efficacy depends on the rationale used to select AC points.

**Methods:**

We developed a method to identify individual patterns of pain and a corresponding set of acupoints (corrAC) based on the relative pressure sensitivity of six abdominal visceral pressure points, i.e., the gastrointestinal (GI) checkpoints (G1–G6). Patients with moderate to severe pain were included and received a single AC treatment following surgery. The visual analog scale (VAS) score, pain threshold, and skin temperature were assessed before AC and at 5 min, 1 h, and 24 h following AC. AC was performed with 1-mm-deep permanent needles.

**Results:**

From April 2021 to March 2022, 72 patients were included in the analysis. Fifty-nine patients received corrAC, whereas 13 received a noncorresponding AC (nonAC) as an internal control. Patients receiving corrAC showed a significant reduction (74%) in pain at 5 min after treatment (*p* < 0.0001) and a significant increase (37%) in the pain threshold (*p* < 0.0001). In this group, a significant increase in skin temperature above G1, G3, G4, and G5 was observed. Patients receiving nonAC showed neither significant pain reduction nor significant changes in pain threshold. The skin above G3 and G4 did not reveal temperature changes.

**Conclusion:**

Checkpoint AC may be an effective tool in postoperative pain therapy after bariatric surgery. Vegetative functional involvement might be associated with pain relief.

**Graphical Abstract:**

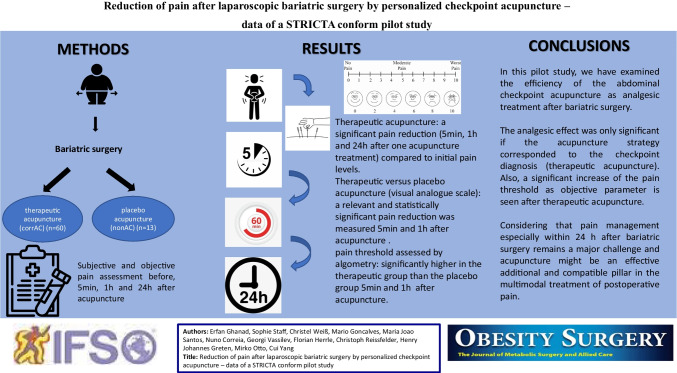

**Supplementary Information:**

The online version contains supplementary material available at 10.1007/s11695-023-06654-8.

## Introduction

Bariatric surgery is a successful method for achieving long-term weight loss among individuals with severe obesity [1]. It is challenging to achieve optimal postoperative pain management for individuals undergoing bariatric surgery, particularly during the first 24 h after surgery [2, 3]. Therefore, a multimodal pain management approach is necessary [4].

Acupuncture may be regarded as a reflex therapy, as it involves eliciting vegetative reflexes via acupoints on the skin. These acupoints are located on meridians, which are neurosensory organs involved in homeostatic vegetative regulation of the body. Stimulating acupoints can lead to functional changes in tissues along the meridian, including the skin, underlying muscles and joints, and other tissues, that are essential for the treatment of various conditions, such as wound pain (e.g., in sutures) or posttraumatic pain. Functional modulation also occurs in symptomatic regions of the viscera, such as peristalsis [5]. Acupuncture (AC) has become an increasingly popular strategy for treating acute and chronic pain [6]. However, the process of selecting acupoints for specific patterns of complaints is essential for the success of AC treatment [7–10] and enhances efficacy [11–13]. Therefore, personalized allocation of AC points should be performed in an effective and rationally accessible theoretical framework [9, 10, 14].

We developed a novel model to evaluate abdominal dysfunction by comparing pressure sensitivity at defined visceral points: Gastro 1–Gastro 6 (G1–G6). The diagnostic usage of pressure points has a long history in surgery as well as in Chinese medicine. The most well-known point for appendicitis in modern medicine is the McBurney point.

**Fig. 1:**
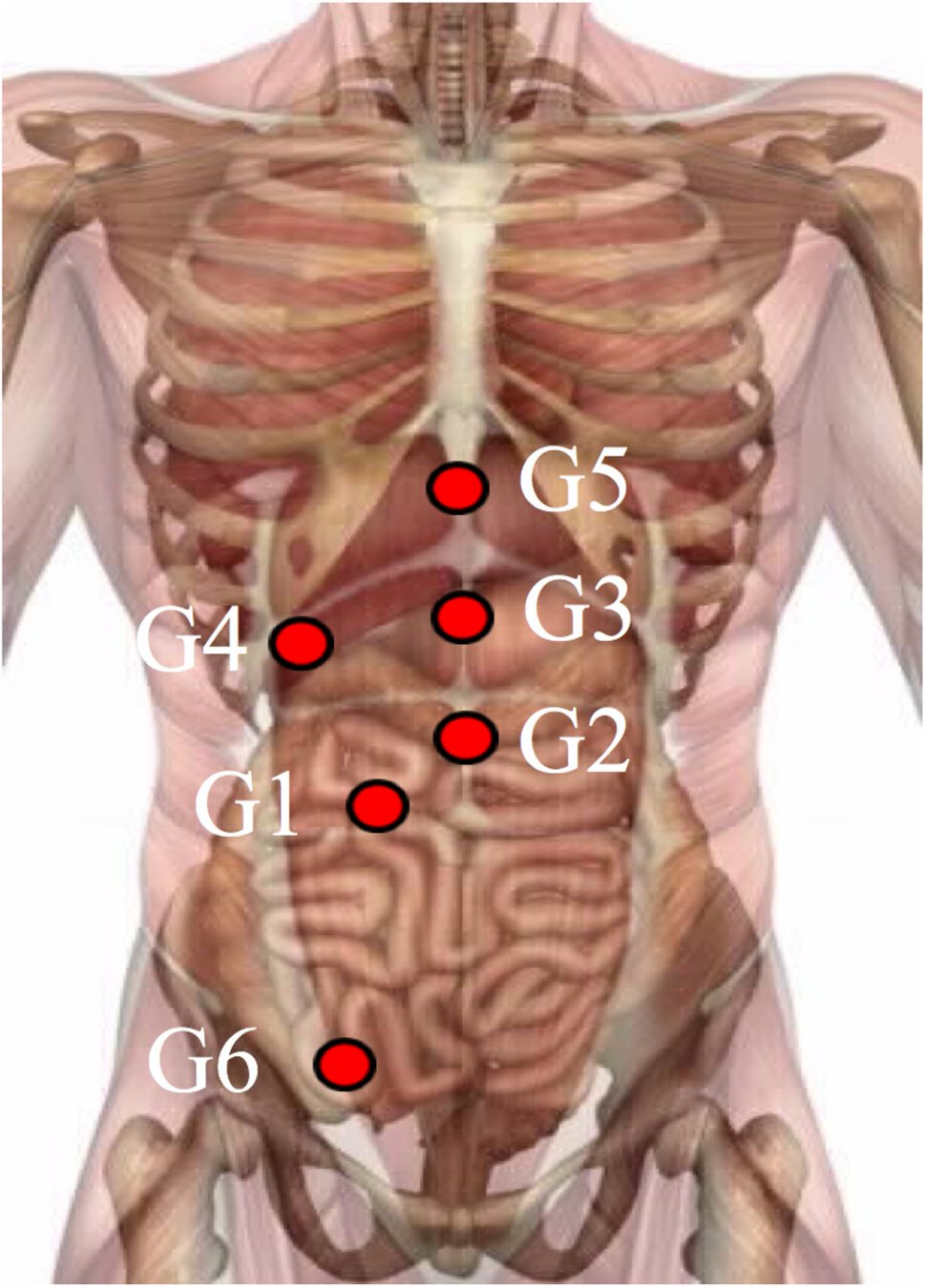
Locations of visceral checkpoints: G1–G6. G1 is located above the sphincter Oddi. G2 is above the pylorus (G2). The gastric fundus (G3) and cardia (G5) are located on the midline. G4 is based subcostal on the midclavicular line (corresponding to the gallbladder). G6 marks the transition from the small intestine to the large intestine

We identified six abdominal points located at key regions of control of gastrointestinal peristaltic movement and digestion (Fig. 1). We also manually compared pressure sensitivity within these visceral pressure points to identify the predominantly painful pressure point (ppp), and a set of suitable acupoints was then selected.

We hypothesize that complex dysfunctional vegetative patterns are connected to these points, which we call G1–G6 syndromes, and that these patterns require the use of a matching set of acupoints to personalize AC therapy, i.e., checkpoint acupuncture. To date, this system is routinely used to treat various abdominal conditions, such as irritable bowel disease and food intolerance, based on the assumption that the complaints are predominantly based on dysfunctional vegetative symptoms. Since GI dysfunctions are often associated with disorders of the autonomic nervous system [16] and pain is moderated by the autonomic nervous system [17], it is conceivable that acupuncture techniques, which are sufficient for treating GI dysfunctions, might also have an impact on postoperative pain treatment.

To verify the applicability of the concept of checkpoint acupuncture in patients after bariatric surgery, we conducted this trial to evaluate its analgesic effect in the postoperative course.

## Methods

### Study Design

This study used a prospective, single-blinded, cohort design to investigate the effects of acupuncture treatment in patients after bariatric surgery. Acupuncture was performed in addition to postoperative standard care, including pain control treatment. Informed consent was obtained prior to enrollment in accordance with a clinical trial protocol approved by the local Ethical Committee. The study was conducted at a university hospital and registered in the German Clinical Trials Register (DRKS00025579).

### Eligibility Criteria

Adult patients who underwent bariatric surgery were screened consecutively. The inclusion criterion was a postoperative pain score of ≥ 3 on a 10-point visual analog scale (VAS). Patients with needle phobia, previous experience with postoperative acupuncture, chronic pain syndrome prior to surgery, polyneuropathy, relevant coagulation disorders, impaired mental state, or poor communication skills were excluded.

### Intraoperative Standard Care

All patients received a standardized anesthesia with a multimodal analgesia technique. Anesthesia was induced with sufentanil (0.3–0.5 mg/kg), propofol (1.5–2.0 mg/kg), and esketamine (0.5 mg/kg) and maintained with continuous infusion of propofol an esketamine. Patients received metamizole and a prophylactic anti-emetic therapy consisting of dexamethasone 8 mg i.v. and granisetron 1 mg i.v. Supplemental intraoperative analgesia was provided using sufentanil or esketamine at the discretion of the attending anesthetist.

All patients underwent surgical site infiltration with 0.25% bupivacaine at the beginning of surgery. A 1-cm skin incision was made 15 cm below the xiphoid paramedian-left. The first 12-mm optical trocar was then inserted through this incision under visual control. The constriction of the pneumoperitoneum was 12 mmHg. Additional 12-mm disposable trocars were inserted in the left upper abdomen at the mammillary line and in the right upper abdomen at the mammillary line. A 5-mm trocar was inserted on the left below the costal arch and anterior axillary line. Using a subxiphoid incision, another 5-mm trocar was set, through which the left liver was lifted ventrally using forceps.

### Surgical Interventions

According to our internal hospital indication guidelines, RYGB was recommended if gastroesophageal reflux disease (GERD) was diagnosed previously. VSG was recommended particularly to patients with a BMI ≥ 50 kg/m2, or to young female patients who desired to have children in the future.

### Standardized Postoperative Recovery Protocol

All patients were monitored in the postanesthesia care unit (PACU) and transferred to a surgical ward as soon as their vital signs were stable. On the surgical ward, they received a scheduled baseline analgesic such as acetaminophen (also known as paracetamol, 1 g every 6 h) or dipyrone (also known as metamizole, 1 g every 6 h). The first-line rescue analgesic was an opioid such as controlled-release oxycodone with naloxone (oral 20 mg/10 mg every 12 h). The second-line rescue analgesic was immediate-release oxycodone (oral 10 mg, maximally every 6 h). Antiemetics were administered on demand. After surgery, clear fluids were allowed if patients denied nausea and vomiting. On the following day, patients started with a fluid diet including clear fluids and protein shakes. They were discharged from the hospital on the second day after surgery if they did not show any signs of complications such as bleeding or leakage of the gastrointestinal tract, tolerated the fluid diet (at least 1 L per day), and had well-controlled pain.

### Visual Analog Scale (VAS)

The intensity of subjective pain—ranging across a continuum from none (0) to an extreme intensity of the pain (10)—was assessed immediately before AC and at 5 min, 1 h, and 24 h after AC.

### Predominant Pressure Point (ppp)

The most sensitive pressure point (ppp) was identified by comparing the sensitivity at all abdominal checkpoints (compare Fig. 1 and Table 1).

**Table 1:** Location of G1–G6 points and their corresponding anatomic structures

G-syndrome	Orientation by Western surface anatomy	Orientation by acupoints	Target region		
G1	At the level of the navel, and the middle of the rectus sheath	St25*	Sphincter Oddi		Sphincter Oddi
G2	On the midline of the abdomen, between G3 and the navel	Ren 10*	Pylorus	Pylorus	
G3	On the midline of the abdomen, in between the navel and the xiphoid process	Ren 12*	Gastric corpus	Gastric corpus	
G4	Under the costal arch, in the mid-clavicular line	Gb 24	Gallbladder	Gallbladder	
G5	Under the xiphoid process	Ren 15	Antrum and His angle	Antrum and His angle	
G6	On the lateral rim of the rectus sheath, on the level of the tendinous inscription below the navel	Between L13-L14	Ileocecal valve	Terminal ileum appr	

Meridians *Gb*, gall bladder; *St*, stomach; *Ren*, ren mai; *, mu points (alarm points) according to Ancient Chinese theory

### Reasoning for AC Selection

The abdominal checkpoints are target regions to be palpated from the exterior to explore the functional state of the corresponding anatomical regions and their structures (see Table 1). Palpation of these target regions is comparable to some clinical examination techniques of Western medicine, such as Murphy’s or McBurney’s sign. To date, abdominal checkpoints are an extension of western palpatory examination routines. Both examples include localization of their target region by certain orientation points of surface anatomy. The anatomic orientation points for the abdominal checkpoints are listed in Table 1.

It is noteworthy that palpation of the depth of the abdominal cavity is uncommon in traditional diagnostics, so to the best of our knowledge, there are no reports on our target regions in the TCM literature. The underlying traditional concepts are not even based on clear anatomic knowledge. Traditional palpatory concepts therefore focus on pulse diagnosis and tender regions of the dermis and subcutis, i.e., the so-called “surface” (defined as skin and meridians). Some of these points are referred to as trigger points or “alarm points” and are of diagnostic value [18, 19].

However, abdominal checkpoints can also be localized via topologic orientation through acupoints, which are abundantly present on the abdomen (Table 1).

The target regions of the visceral G points were first observed by medical doctors in patients with a variety of functional abdominal problems in an outpatient clinic. They observed that the pressure sensitivity of these target regions may be an important clue for the individual discrimination of disease patterns and the individual selection of acupoints [20]. These acupuncture strategies are based on a theory from Chapter VI of the Su Wen called The Three Principles [21]. In this sense, augmented pressure sensitivity in the G1 suggests treatment via the meridians of the pivotal ore hinge principle, in G3 to treatment of the so-called opening principle as described in Su Wen and other literature [9, 20, 21]. They refer to the teaching of the six “meridians” that are a major concept within a classic scripture [22].

A few exploratory pilot studies were performed on the measurable physiologic effects of the G point concept as master’s theses; the results of which were summarized in a published paper [15]. Surgeons of our hospital suggested the probatory usage of these points after abdominal surgery. The sets of acupoints chosen for the G Syndromes were adapted to the surgical context over time, as surgery-induced symptoms are clinically distinct from mere functional gastrointestinal disease. The adopted sets of acupoints used for this study are listed in Table 2.

**Table 2:** G-syndromes and corresponding standardized sets of acupoints

G-syndrome (ppp)	Standardized set of acupoints
G1	Right: Sj 3; left: Pc 5, Gb 41; both sides: Sp 6
G2	Right: Li 3; left: St 25, St44; and Ren 10
G3	Right: Li 3; left: Lu 5, Lu 7, St16, Sp 6, St 25, St 44; and Ren 17
G4	None of the patients showed a G4-syndrome
G5	Right: Pc 6, Li 2, LI 10, Sj 3, Left: St16, St 44, Gb 41, both sides: Sp 16, and Ren 17
G6	Right: Si 6, Bl 62; left: He 3 both sides: Sp 6

*Ppp* predominant pressure point, Meridians *Ht* heart; *Lu* lung; *Pc* pericardium; *Si* small intestine; *LI* large intestine; *Sj* triple burner; *Lv* liver; *Ki* kidney; *Sp* spleen; *Bl* urinary bladder; *Gb* gall bladder; *St* stomach; *Ren* ren mai

### Pressure Algometry

Using an algometry device (PCE-PTR 200 N), the pressure was slowly increased on the ppp until it was perceived as uncomfortable. This pressure was defined as the mechanical pain threshold. [23] Algometry was performed immediately before and at 5 min, 1 h, and 24 h after AC.

### Skin Temperature

Contact-free temperature measurement on the abdominal skin was performed (infrared thermometer NT17, model: Domotherm Free) as an indicator of change in microcirculation. The temperature was measured immediately before AC and at 5 min, 1 h, and 24 h after AC.

### Needling Technique

For AC, a commercially available set of permanent needles with an in-built prefabricated needle applicator (Sedatelec ASP Original Classic steel needle) was used to ensure consistent insertion pressure, depth, and stimulation intensity. These needles limit the depth of insertion into the skin to 1 mm.

### Corresponding Personalized Checkpoint Acupuncture (corrAC)

Before AC, the pressure sensitivity of G1–G6 was manually compared to determine the ppp. If the most pressure-sensitive point was, e.g., above G1, a G1 syndrome was diagnosed. Based on the diagnosis, a standardized set of acupoints was needled to conduct the corresponding personalized checkpoint acupuncture (corrAC) (see Table 2). Patients received rescue analgesics if their pain persisted after acupuncture therapy.

### Noncorresponding AC (nonAC)

We also investigated the potential therapeutic effect of other AC strategies; for example, patients with G5 syndrome were treated with the G3 regimen, and those with G3 syndrome were treated with the G1 regimen. Patients were blinded to the kind of AC regimen we referred to as “noncorresponding AC” (nonAC). The acupoints used within these strategies are also commonly used for abdominal complaints, although they have been used to treat other types of G-syndromes. They may serve as internal controls. Patients received rescue analgesic if their pain persisted after acupuncture therapy.

### STRICTA Criteria

All data were collected in accordance with the STRICTA criteria [24].

1. Clear acupuncture rationales were followed, in accordance with the checkpoint concept (G-points) described in previous papers, based on reflections apparent in the Shang Han Lun [22], a traditional Chinese medicine treatise that Zhang Zhongjing compiled before 220 AD. All patients were diagnosed accordingly.
2. Details of needling included an identical number and depth of needling with identical needle retention times of 5 min, 1 h, and 24 h. There was no needle stimulation in the verum group and controls. Diameter, length, pressure of insertion, and depth of insertion were unchangeably identical due to the constructive features of Sedatelec permanent needles and their respective applicator. We did not seek subjective needle sensation (de qi) or other individual responses.
3. The treatment regimen was identical in all groups, as there was only one treatment after surgery, with permanent needles remaining over the whole observation period. Oral communication was restricted to a minimum in a stress-avoiding atmosphere.
4. No further interventions of Chinese medicine (CM) were administered, and no further lifestyle advice was given. Patients were uniformly informed 1 day before treatment using a standardized information and explanation sheet. The setting and context of treatment were identical in terms of hospital rooms, time of onset of therapy, nutrition, and fluid intake.
5. All practitioners had performed acupuncture on a daily basis for more than 5 years.
6. Control or comparator interventions. The rationale for the control in the context of the current research question is that the strictest control intervention is to needle a set of acupoints with identical depth, intensity, and number instead of placebo needles or non-acupoints or any other type of sham acupuncture. For this purpose, we used two sets of acupoints that are frequently used for abdominal complaints but empirically when G1 or G6 are the dominant visceral pressure sensitive points within an individual. This strategy was used with the aim of identifying reliable or unreliable sets of control points for further usage based on STRICTA guidelines.

### Statistical Analysis

For statistical analysis, SAS software, release 9.4 (SAS Institute, Cary, NC, USA) was used. Quantitative variables are presented as the mean ± standard deviation; for qualitative factors, absolute and relative frequencies are given. To compare two groups, Fisher’s exact test or a 2-sample *t* test was used, as appropriate. Repeated-measures ANOVA was used to investigate changes over time. Furthermore, a two-way repeated measurements ANOVA was performed to analyze the impact of measurement time and a group factor on a quantitative outcome simultaneously. Furthermore, the Scheffé post hoc test was conducted to perform pairwise comparisons. These analyses were performed with the SAS procedure PROC MIXED with time and group as fixed factors and patients’ ID as a random factor.

GraphPad Prism was used to create the figures. The result of a statistical test was considered significant when *p* < 0.05 (*).

## Results

### Demographics

Between April 2021 and April 2022, 72 patients were included in the study. Sixty patients (83%) were females, and 12 patients (17%) were males. The mean age was 42.6 ± 11.4 years (range: 23–66). **A**mong the included patients, 53 underwent laparoscopic Roux-en-Y gastric bypass (RYGB) and 19 underwent laparoscopic vertical sleeve gastrectomy (VSG). Men were more likely to undergo VSG than RYGB. The VSG group had a higher weight and BMI than the RYGB group, as shown in Table 3.

**Table 3:** Baseline patient characteristics

	RYGB (*n* = 53)	VSG (*n* = 19)	*p* values RYGB-VSG
Female (*n*, %)	50 (94%)	10 (53%)	0.0001
Age (years)	43.1 ± 11.5	41.3 ± 11.1	0.5707
Weight (kg)	119.9 ± 19.5	151.8 ± 31.9	0.0004
Preoperative BMI (kg/m^2^)	44.6 ± 9.5	51.6 ± 8.6	0.0063

*RYGB*, Roux-en-Y gastric bypass; *VSG*, vertical sleeve gastrectomy; *BMI*, body mass index. Qualitative factors are presented by their absolute and relative frequencies; for quantitative variables, mean ± standard deviation is given

The average length of stay was comparable in both groups (2.3 ± 1.1 days in the corrAC group vs. 2.6 ± 1.7 days in the nonAC group; *p* = 0.57). In each group, one postoperative complication was noticed: one patient had metamizole-induced leukopenia of low severity. One patient in the corrAC group showed an anastomotic leaking of the pouch, which was successfully treated with endoluminal vacuum therapy.

### Distribution of G-Syndromes

Out of 72 patients, 52 (72%) showed G3 syndrome. In 13 patients (18%), G5 was the predominantly pressure-sensitive point. Five patients (7%) had G2 syndrome, one (1%) had G1 syndrome, and one (1%) had G6 syndrome.

### Pain Levels After G-Point Corresponding AC Compared to Noncorresponding AC

**Fig. 2:**
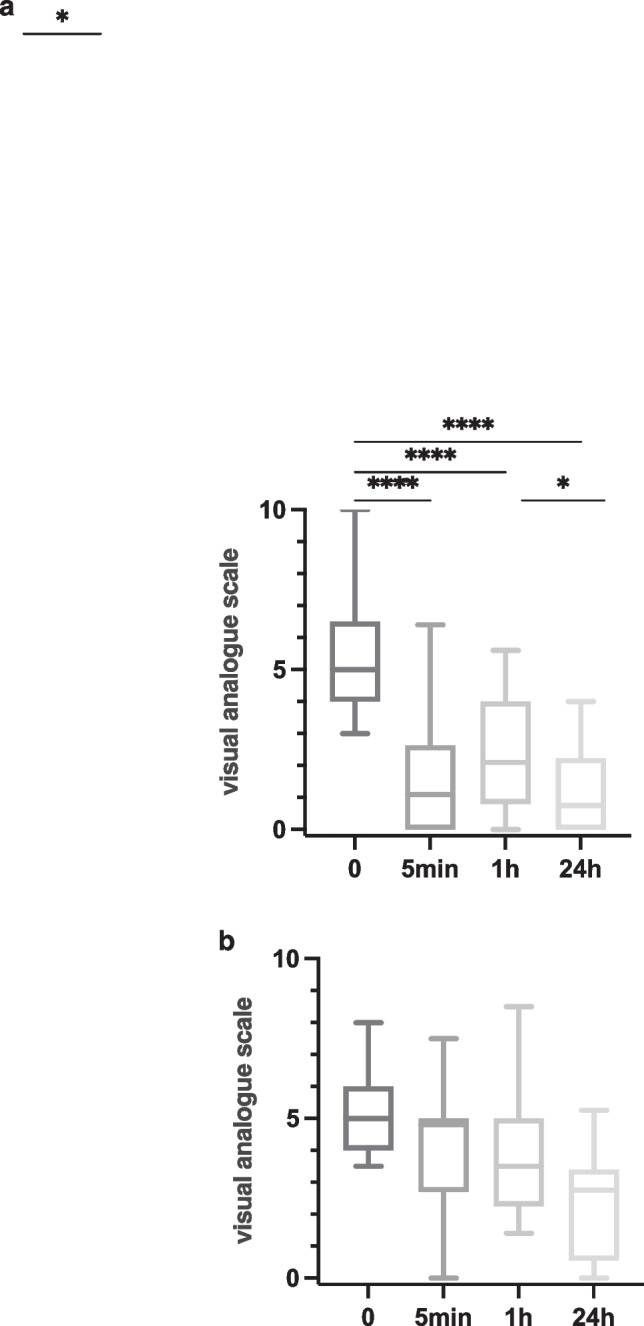
**a** VAS after corrAC: Significant pain reduction after 5 min, 1 h and 24 h compared to initial VAS before AC (three asterisks (****): each *p* < 0.0001). Furthermore, a significant pain reduction between 1 and 24 h was measured (one asterisk (*): *p* = 0.0466). Minimum and maximum VAS levels shown through box-and-whisker plots. **b** VAS after nonAC. No significant pain reduction was observed at any time point. Minimum and maximum pain thresholds are shown though box-and-whisker plots

The mean VAS (corrAC vs. nonAC: 5.4 ± 1.6 vs. 5.1 ± 1.5) was not significantly different (*p* = 0.5162) between the groups before AC. Since the two-way ANOVA including the factors time and group (corrAC vs. nonAC) showed a significant interaction term (*p* = 0.0008), separate one-way ANOVAs were performed for both groups. For patients who received a G-point-corresponding AC (corrAC), significant changes over time were observed (*p* < 0.0001, Table 4, Fig. 2a). The Scheffé’s test revealed a significant pain reduction from baseline to each time point after AC (each *p* < 0.0001), as well as an increase from 5 min to 1 h (*p* = 0.0203) followed by a significant reduction from 1 to 24 h (*p* = 0.0466). For patients with noncorresponding AC, one-way ANOVA showed nonsignificant results (*p* = 0.0512, Table 4, Fig. 2b). The Scheffé’s test for pairwise comparisons also revealed no significant differences.

**Table 4:** VAS scores for corresponding and noncorresponding AC

	Corresponding (*n* = 59)	Non-corresponding (*n* = 13)
Baseline	5.4 ± 1.6	5.1 ± 1.5
5 min	1.6 ± 1.8	4.2 ± 2.1
1 h	2.4 ± 1.7	4.1 ± 1.9
24 h	1.1 ± 1.2	2.2 ± 1.8
Average reduction from baseline to 5 min	74%	16%
Average reduction from baseline to 1 h	57%	23%
Average reduction from baseline to 24 h	81%	59%

**Fig. 3:**
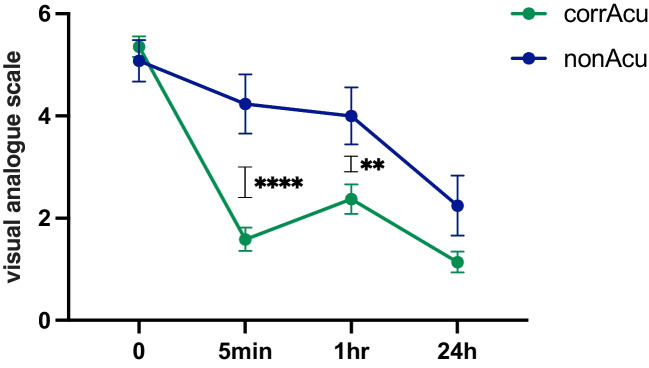
Comparison of the pain development of the G-point corrAC (green) and nonAC (blue) groups. No significant difference in VAS scores was observed at the beginning (*p* = 0.5162) or after 24 h (*p* = 0.0822). However, after 5 min (*p* = 0.0003) and after 1 h (*p* = 0.0016), a clinically relevant and statistically significant difference between the two groups was observed

Comparing the pain level developments after AC for both the corrAC and nonAC groups, the former group showed significantly lower pain levels, as shown in Fig. 3.

### Pain Levels Considering Different Surgical Procedures—RYGB and VSG

Before AC, the mean VAS score for all patients was 5.3 ± 1.6 [3–10], indicating moderate to severe pain. Patients who underwent RYGB had a similar pain level as those who underwent VSG (5.2 ± 1.6 vs. 5.6 ± 1.5, *p* = 0.3345).

CorrAC led to a significant pain reduction for RYGBs and sleeve gastrectomies (Table 5). Two-way ANOVA revealed significant changes in VAS scores over time (*p* < 0.0001) but no difference between the RYGB and VSG (*p* = 0.5673) and no interaction between treatment and time (*p* = 0.4657), indicating that changes in VAS scores over time were similar in both treatment groups. The Scheffé test revealed a significant reduction in pain from baseline to 5 min (*p* < 0.0001), followed by a significant increase from 5 min to 1 h (*p* = 0.0019) and then a significant reduction again from 1 to 24 h (*p* = 0.0111).

**Table 5:** VAS scores for RYGB and VSG

	RYGB (*n* = 53)		VSG (*n* = 19)	
Baseline	5.2 ± 1.6		5.6 ± 1.5	
	AC	Controls	AC	Controls
5 min	1.6 ± 1.8	4.8 ± 1.9	1.5 ± 2.0	2.9 ± 2.1
1 h	2.3 ± 1.7	3.9 ± 1.4	2.5 ± 1.8	4.5 ± 3.0
24 h	1.1 ± 1.2	2.1 ± 2.0	1.3 ± 1.6	2.6 ± 1.5
Average reduction from baseline to 5 min	73%	8%	74%	33%
Average reduction from baseline to 1 h	56%	24%	61%	20%
Average reduction from baseline to 24 h	80%	61%	83%	55%

### Pressure Pain Threshold Before and After AC

This subjective pain was objectified by evaluation of the pain threshold using algometry. The initial average pain threshold was reached at 15.7 ± 7.0 N. The initial average pain threshold was 15.7 ± 7.0 N. Before intervention, no difference in the pain threshold was observed between the groups receiving either corresponding or noncorresponding treatment (15.6 ± 7.4 N vs. 15.7 ± 5.3 N, *p* = 0.9694; see Table 6). Due to the significant interaction between group and time (*p* = 0.0324, supplementary materials Fig. 1a), two separate one-way ANOVAs were conducted. In the G-point corresponding group, AC led to a significant increase in the pressure pain threshold 5 min after acupuncture to 20.6 ±  11.1 N (*p* < 0.0001, supplementary materials Fig. 1b). This result corresponds with an equivalent increase in pain threshold by 37% on average. In the nonAC group, AC showed no relevant effect on the pressure pain threshold throughout the period of observation (*p* = 0.1138, supplementary materials Fig. 1c).

**Table 6:** Algometry for corresponding and noncorresponding AC

	Corresponding (*n* = 59)	Non-corresponding (*n* = 13)
Baseline	15.6 ± 7.4	15.7 ± 5.3
5 min	20.6 ± 11.1	15.4 ± 6.1
1 h	16.5 ± 7.5	12.2 ± 4.6
24 h	18.4 ± 9.1	13.3 ± 5.9

### Small Skin Temperature Changes After AC

To investigate changes over time, repeated measurements ANOVA was conducted for each area and AC group, along with the Scheffé test for pairwise comparisons.

After the intervention, the corrAC group showed a slight but significant increase in temperature levels above the areas of G1, G3, G4, and G5 24 h after intervention (supplementary materials Table 1a). The nonAC treatment also led to a slight and significant increase in temperature at G5 but not at G1, G3, and G4 (supplementary materials Table 1a and 1b). Temperature elevation in G1, G3, and G4 might therefore be connected to pain relief by changes in microcirculation at these points as a sign of different types of vegetative reactions to AC.

## Discussion

In this pilot study, we examined the efficiency of abdominal checkpoint acupuncture as analgesic treatment after bariatric surgery. Our results clearly demonstrated a marked pain reduction after AC, which lasted for at least 24 h after the treatment. We also confirmed that this analgesic effect was only significant if the therapeutic acupuncture strategy corresponded to the G point diagnosis.

The abdominal checkpoints shown in Fig. 1 may be regarded as a tool to identify useful AC point strategies addressing patient discomfort. For most participants, G3 (72%) and G5 (18%) were the most sensitive checkpoints. This phenomenon might have an anatomical basis. G3 corresponds to the fundus, and G5 corresponds to the cardia. During laparoscopic vertical sleeve gastrectomy (VSG) and Roux-en-Y gastric bypass (RYGB), clearing the top of the fundus and angle of His (also called the esophagogastric angle) is essential. Both the fundus and the cardia are involved in this surgical step. Moreover, VSG is associated with increased intragastric pressure, which further enhances the challenge to the cardia.

Acupuncture is often seen as a vegetative reflex therapy in which reflexes are induced via specific reflex points (acupoints) on the skin [7–9, 25, 26], modulating the autonomous nerve system. The appropriate selection of acupoints should thus be dependent on the clinical presentation itself. The underlying physiological basis has long been debated. One hypothesis is that long-distance acupuncture effects operate partly through somato-autonomic reflexes, leading to modification of sympathetic and parasympathetic pathways [27]. According to our data, the acupoints used in this syndrome are able to significantly reduce subjective and objective pain, whereas the stimulation of other sets of acupoints did not result in substantial reductions in. Certain psychological factors such as anxiety and depression seem to have an influence on both the experience of pain and the analgesic treatment outcome [28, 29]. Few studies support the effectiveness of acupuncture as a treatment for depression and anxiety disorders [30, 31]. This might further enhance the analgesic effect in our trial.

Our data were acquired by rigorous application of the STRICTA criteria [32] for reporting on clinical acupuncture. However, these data were obtained in a pilot study in the context of the development of the final study design. We evaluated pain not only by subjective visual analog scales but also by objectively measurable physical parameters such as skin temperature and pressure algometry [33, 34]. Hence, we conclude that near-placebo or suggestive effects do not explain the significant pain reduction of the patients. However, the treatments were not yet randomized, and acupuncture was only single-blinded.

Furthermore, this finding can be explained by the skin temperature changes. Skin temperature above G3 and G4 was slightly elevated only at the G point corresponding to AC of G3 syndrome, whereas treatment by other strategies would not elevate this skin temperature above these points and had no significant pain-relieving effect. We hypothesize that this increase in microcirculation after AC may support the theory that this is part of the vegetative reflex mechanisms underlying the pain-reducing effect. Further studies are needed to investigate this phenomenon.

Considering that pain management, especially within 24 h after bariatric surgery, remains a major challenge [2, 3], AC might be effective as an additional and compatible pillar in the multimodal treatment of postoperative pain.

To the best of our knowledge, our data represent the first usable and feasible strategy of somatic acupuncture in postoperative pain of bariatric patients [35].

## Conclusion

Our data show that abdominal checkpoint acupuncture may present an effective complementary tool for postoperative pain management even within enhanced recovery pathways. Using the abdominal pressure points (G1-G6), postoperative pain can be systematically personalized and addressed after surgery. Breaking down the complexity of the functional traditional diagnosis used for acupuncture to a few abdominal pressure points may lead to rational access to effective acupuncture strategies.

Based on our results, we developed a triple-blinded RCT to evaluate the effectiveness of acupuncture after abdominal surgeries.

## Supplementary Information

Below is the link to the electronic supplementary material.

Supplementary file1 (DOCX 4280 KB)
