# Supplementary material for: Reduction of Pain After Laparoscopic Bariatric Surgery by Personalized Checkpoint Acupuncture—Data of a STRICTA Conform Pilot Study
Source: Obes Surg. 2023 May 29;33(7):2176–85. doi: 10.1007/s11695-023-06654-8 (PMC10289919; doi:10.1007/s11695-023-06654-8)
Supplement: Supplementary file 1 — Supplementary file1 (DOCX 4280 KB) [file 11695_2023_6654_MOESM1_ESM.docx]

**SUPPLEMENTARY MATERIAL**

**Figure 1a** Development of the pain threshold before (p=0.9624) and 5 min (p=0.0284), 1 h (p=0.0303) and 24 h (p=0.0673) after AC. Comparing both groups: corrAC vs nonAC. P values were calculated by using Satterthwaite t- test.

**
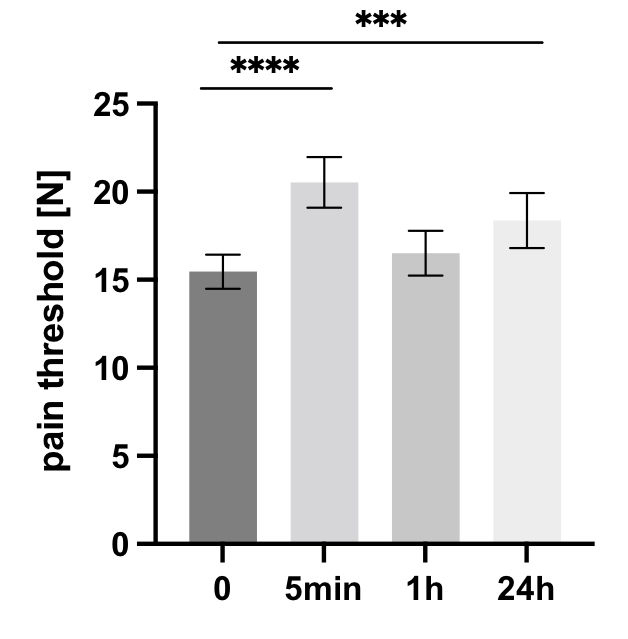
**

**Figure 1b** corrAC: Development of the pain threshold before and 5 min, 1 h and 24 h after AC. A significant pain threshold increase was seen 5 min after AC (p < 0.0001). Also, the difference between baseline and 24 h was significant (p = 0.0028). Other differences were not significant.

**
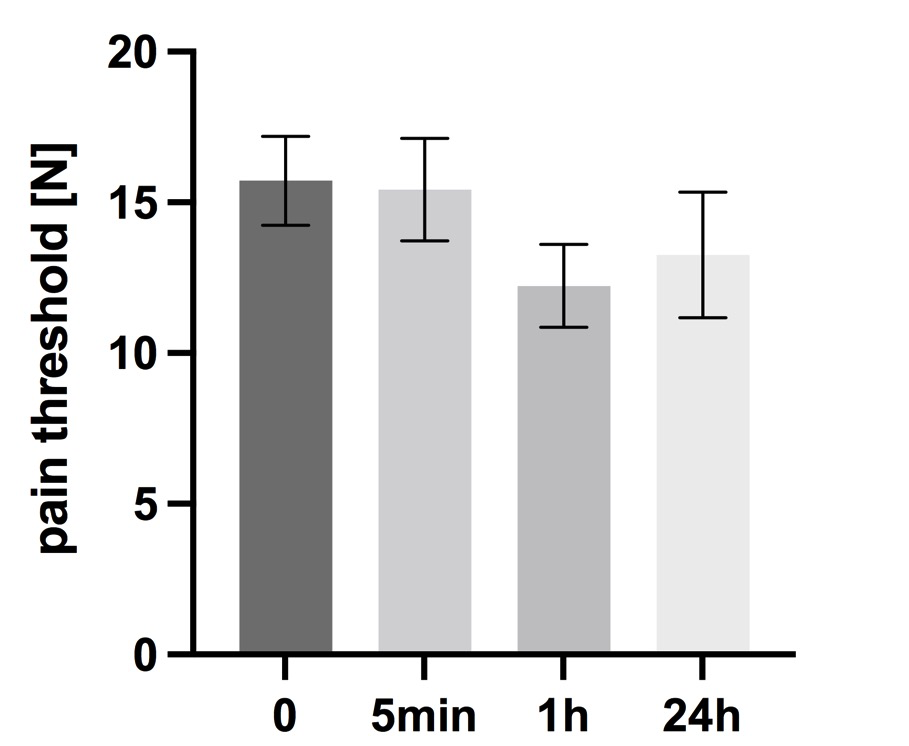
**

**Figure 1c** nonAC: Development of the pain threshold before and 5 min, 1 h and 24 h after AC. No significant changes were seen (p = 0.1138).

**Table 1a** Skin temperatures for corresponding AC.

| **Area** | **baseline** | **5 min** | **1 hour** | **24 hours** | **p value ANOVA** | **p value Scheffé 0 – 24h** |
| --- | --- | --- | --- | --- | --- | --- |
| G1 | 36.9 ± 0.4 | 36.8 ± 0.3 | 37.0 ± 0.4 | 37.2 ± 0.6 | 0.0070 | 0.0320 |
| G2 | 36.8 ± 0.3 | 36.8 ± 0.3 | 37.0 ± 0.4 | 37.0 ± 0.6 | 0.1060 | --- |
| G3 | 36.8 ± 0.3 | 36.8 ± 0.3 | 36.9 ± 0.3 | 37.0 ± 0.4 | 0.0084 | 0.0224 |
| G4 | 36.9 ± 0.3 | 36.8 ± 0.2 | 36.9 ± 0.3 | 37.2 ± 0.5 | 0.0002 | 0.0005 |
| G5 | 36.9 ± 0.4 | 36.8 ± 0.3 | 36.9 ± 0.3 | 37.2 ± 0.5 | 0.0009 | 0.0063 |

**Table 1b** Skin temperatures for non-corresponding AC

| **Area** | **baseline** | **5 min** | **1 hour** | **24 hours** | **P value ANOVA** | **p value Scheffé 0 – 24h** |
| --- | --- | --- | --- | --- | --- | --- |
| G1 | 36.9 ± 0.2 | 37.0 ± 0.4 | 36.7 ± 0.4 | 36.9 ± 0.2 | 0.2813 | --- |
| G2 | 36.9 ± 0.2 | 36.9 ± 0.4 | 36.7 ± 0.5 | 36.9 ± 0.2 | 0.5263 | --- |
| G3 | 36.9 ± 0.2 | 36.8 ± 0.1 | 36.8 ± 0.1 | 36.8 ± 0.1 | 0.6209 | --- |
| G4 | 36.8 ± 0.2 | 36.9 ± 0.3 | 36.8 ± 0.2 | 37.2 ± 0.7 | 0.1953 | --- |
| G5 | 36.9 ± 0.2 | 36.8 ± 0.2 | 36.8 ± 0.3 | 37.3 ± 0.4 | 0.0115 | 0.0378 |
